# Friday: Fish Day

**DOI:** 10.5334/jbr-btr.1047

**Published:** 2016-02-12

**Authors:** Pieter Hoste, Koenraad Mortele, Marc Lemmerling, Dirk Dewilde, Adelard De Backer

**Affiliations:** 1 UZ Ghent, BE; 2 BIDMC Harvard, US; 3 AZ Sint-Lucas Gent, BE

**Keywords:** abdominal abcess, perforation, foreign body, complications, peritonitis, risk factors

## Abstract

A 78-year-old man presented with diffuse abdominal pain, localized peritonitis and raised inflammatory markers. CT revealed an mesentery abcess with a linear high density structure in continuity with the adjacent small bowel lumen. Laparoscopy showed a perforation by a fish bone. Gastrointestinal tract perforation by foreign body ingestion is rare complication (1%). There are some risk factors and prefered locations of perforation. Time interval between ingestion and complication can vary extremely.

## Case Report

A 78-year-old man presented with diffuse abdominal pain and nausea without vomiting for two days. Patient had his last bowel movement three days before. Physical examination confirmed diffuse abdominal pain and showed rebound tenderness in the periumbilical region suggestive of localized peritonitis. Laboratory analysis showed an elevated C-reactive protein (CRP) level of 261 mg/L (normal value < 5 mg/L) and elevated leucocyte count of 15,400/µL (normal values 3,400–9,800/µL). Plain film of the abdomen (not shown) demonstrated dilatation of a few small bowel loops in the lower abdomen with air fluid levels and absence of pneumoperitoneum. Computed tomography (CT) of the abdomen, after intravenous administration of iodinated contrast material, showed an inflammatory mass in the mesentery of the small bowel with a central air fluid level. A linear high-density structure was noted within the inflammatory mass and was in continuity with the adjacent small bowel lumen (Figure A and B, arrow). Sagittal (Figure C, arrow) reformatted images confirmed a foreign body, suggestive of a fish bone, perforating the small bowel wall and with abscess formation. Patient admitted to ingestion of a fish bone eight days earlier. Subsequently, laparoscopy was performed. Patient was successfully treated with abscess drainage, fish bone removal, segmental resection of the inflamed bowel segment with an ileo-ileal anastomosis.

**Figure 1:**
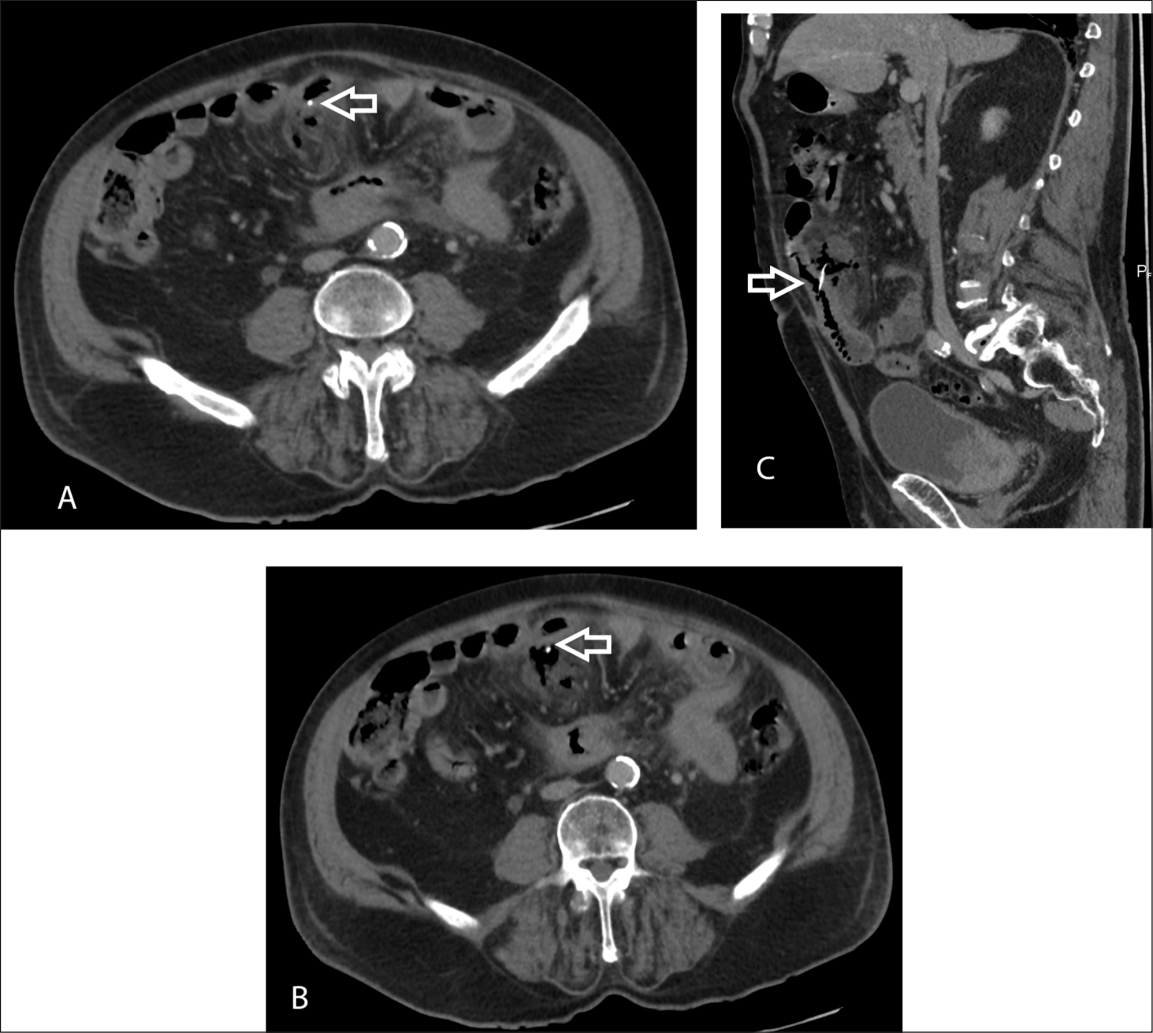
Axial (A and B) and sagittal (C) reformations shows linear high-density structure (arrow) within the inflammatory mass and in continuity with the adjacent small bowel lumen.

## Comment

Ingestion of foreign bodies, especially fish bones, is not an uncommon finding. In most cases, the ingested foreign body passes through the gastrointestinal tract within one week without causing complications. Gastrointestinal tract perforation as a consequence of foreign body ingestion is a relatively rare complication with a reported incidence of 1%. People at increased risk for foreign body ingestion are prisoners, psychiatric patients, and alcohol and drug abusers, but also includes persons at the extremes of their life, persons who eat rapidly, and a set of selected professions (e.g. carpenters and dressmakers). Wearing dentures is a well-known risk factor because it reduces tactile sensation on the palatal surface. In our case, the patient was of old age and was wearing dentures.

Time interval between ingestion of the foreign body, complication and onset of clinical symptoms may vary between days, months or even years, making the diagnosis based on clinical findings and anamnesis very difficult.

CT is the examination of choice for the detection of perforating foreign bodies. Even though perforation may occur in all segments of the bowel, the most common locations are regions of acute angulation, such as the ileocecal and rectosigmoid junctions. CT shows the perforated region as an intestinal segment with thickened wall, adjacent fatty stranding with or without abscess, localized pneumoperitoneum or an associated intestinal obstruction. However, the final diagnosis of bowel perforation caused by foreign body is not possible solely based on these nonspecific findings. Only direct visualization of the foreign body surrounded by inflammation or free air may allow for an accurate diagnosis [1].

In our case, the confident diagnosis of a dense foreign body, representing a fish bone, with perforation of the small bowel and complicated by abscess formation was based on clinical history and CT findings. Final diagnosis was confirmed and treated by laparoscopy.

## Competing Interests

The authors declare that they have no competing interests.
